# Kale supplementation during high fat feeding improves metabolic health in a mouse model of obesity and insulin resistance

**DOI:** 10.1371/journal.pone.0256348

**Published:** 2021-08-25

**Authors:** Samnhita Raychaudhuri, Si Fan, Olivia Kraus, Md. Shahinozzaman, Diana N. Obanda

**Affiliations:** 1 Department of Nutrition and Food Sciences, University of Maryland, College Park, MD, United States of America; 2 College of Computer, Mathematical and Natural Sciences, University of Maryland, College Park, MD, United States of America; Universidade do Estado do Rio de Janeiro, BRAZIL

## Abstract

Cruciferous vegetables have been widely studied for cancer prevention and cardiovascular health. Broccoli is the cruciferous vegetable whose phytochemistry and physiological effects have been most extensively studied. Kale (*Brassica oleracea* var. acephala) appears on lists of ‘healthiest, nutrient dense foods’ but, there is paucity of data on kale as a functional food. In a 12-week study, we tested the effect of curly green kale on high fat diet (HFD) induced obesity and insulin resistance, lipid metabolism, endotoxemia and inflammation in C57BL/6J mice fed isocaloric diets. Kale supplementation did not attenuate HFD diet induced fat accumulation and insulin resistance (P = ns; n = 9) but, it lowered serum triglycerides, low density lipoprotein (LPL) cholesterol and prevented HFD induced increases in systemic endotoxemia and inflammation (serum LPS and Ccl2) (P<0.01; n = 9). In adipose tissue, kale enhanced the expression of genes involved in adipogenesis (P<0.01; n = 9), reduced the appearance of histologic markers of inflammation, downregulated both the gene expression and protein expression of the adipose tissue specific inflammation markers CD11c and F4/80 (P<0.001; n = 9) and reduced the gene expression of a battery of chemokine C-C motif ligands (Ccl2, Ccl6, Ccl7, Ccl8, Ccl9) and chemokine C-C motif receptors (Ccr2, Ccr3, Ccr5). We conclude that kale vegetable protects against HFD diet induced dysfunction through mechanisms involving lipid metabolism, endotoxemia and inflammation.

## Introduction

Dietary induced obesity and its comorbidities have a high societal and economic cost. Due to their physiological benefits, functional foods that provide health benefits beyond the provision of nutrients, have the capability to lower the cost of healthcare [1]. Functional foods represent one of the most intensively investigated and widely promoted area in health sciences today [1, 2]. Diets rich in cruciferous vegetables are associated with lower risk of cancer, inflammation, diabetes, cardiovascular health, neurodegenerative disorders and ocular disorders. Cruciferous vegetables have been shown to have potent inhibitory activities against cancer cell lines [3, 4]. Broccoli is the cruciferous vegetable whose phytochemistry and physiological effects have been most extensively studied and reviewed. Furthermore, most dietary supplements with cruciferous vegetable phytochemicals are made from broccoli [3, 4]. However, a vegetable that is often on the lists of ‘the most healthy foods’ is kale (*Brassica oleracea* var. acephala). Kale has been cultivated for centuries and has been part of many traditional meals, especially in the Mediterranean area. Easily cultivated with resistance to extreme weather, kale has recently become popular in the United States and its production has been steadily growing. Cultivation significantly rose from 3,994 to 6,256 harvested acres in the USA between 2007 to 2012 [5]. It is widely sold as fresh or frozen produce and is common on restaurant menus’.

As with other cruciferous vegetables, the positive effect of kale is attributed to phytochemicals such as the sulphur containing indolic glucosinolates and aliphatic glucosinolates, polyphenols like the flavonoid glycosides of quercetin, kaempferol and isorhamnetin and carotenoid groups [6–11]. While kale and its phytochemicals has been extensively studied for its effects on cancer [12, 13] and cardiovascular health through reduction of cholesterol levels [14, 15], its effects on fat accumulation and insulin resistance or prediabetes and overt diabetes are less clear. Although there are claims on the benefits of kale on type 2 diabetes, there is no solid data to support such claims. Obesity induced insulin resistance caused by accumulation of certain lipid metabolites from excess dietary lipids is a key pathophysiologic feature of type 2 diabetes mellitus. The excess dietary lipids also precipitate in inflammation which also contributes to the risk of insulin resistance, type 2 diabetes and other health consequences linked to obesity. Progression of insulin resistance into overt type 2 diabetes can be prevented or delayed by timely intervention.

In a previous study, we showed that kale increases the diversity of the gut microbiota, increases the representation of beneficial bacterial families associated with health and decreases bacterial families associated with inflammation [16]. In this follow-up study, we focus on the effects of kale on HFD induced fat accumulation, insulin resistance, endotoxemia, lipid metabolism and, further test inflammation using a PCR array for differential expression of 84 genes associated with inflammation. We formulated the control low fat diet (LFD) and control HFD to be isocaloric to the diet containing kale vegetable and tested the efficacy of the vegetable as (i) a prevention strategy for effects of a HFD when used from the beginning of the study or (ii) reversing HFD induced changes by including it in the diet after obesity is induced. We report on effects of the vegetable on body weight, fat accumulation in the liver and adipose tissue, systemic insulin resistance, endotoxemia and the expression of genes involved in adipogenesis, lipogenesis and inflammation in adipose tissue.

## Materials and methods

### Acquisition of plant sample and nutrient composition analysis

The stem and leaves of curly green kale vegetable obtained from the farmers market at the University of Maryland, was chopped, oven dried (50°C) for one day and powdered using a food grinder. The sample was subjected to nutrient composition analysis before incorporation into the diets. The dietary preparation, animal study procedures, and analytical procedures for tissues were as previously shown [17] and are briefly described in the following sections. Results of nutrient analysis by the Association of Analytical Chemists (AOAC) methods for calorific value and proximate analysis: moisture, ash, lipid, protein, carbohydrate, soluble fiber, insoluble fiber and fatty acid profile are shown in S1 and S2 Tables.

### Study diets

We formulated three diets based on the same total amount of calories (3961 Kcal per kg) with the same amount of sucrose and protein. However The LFD control with 10% fat, HFD control with 45% fat and the HFD containing 9% kale (HFKV) as shown in a previous study [16] (Table 1). This dose was arbitrary not based on any prior study. The HFD and HFKV were formulated to be isocaloric at 4.62 Kcal/g while the LFD had 3.73 Kcal/g. The LFD composition is very similar to the AIM-93M mature diet for maintenance, except it had both lard and soybean oil as part of the fat source. The Calories per gram are the same as in AIM -93-M. The composition of diets formulated is shown in Table 1.

**Table 1:** Diet formulation.

	LFD	HFD	HFKV
**Ingredient (g)**			
Casein	200	200	191.88
L-Cystine	3	3	3
Corn starch	452.2	72.8	48.28
Maltodextrin 10	75	100	100
Sucrose	172.8	172.8	172.8
Cellulose	50	50	13.92
Soybean oil	25	25	23.46
Lard	20	177.5	177.5
Mineral Mix	10	10	10
Di calcium phosphate	13	13	13
Calcium carbonate	5.5	5.5	5.5
Potassium citrate	16.5	16.5	16.5
Vitamin mix	10	10	10
Choline Bitartrate	2	2	2
kale dried powdered shoot	0	0	82
**TOTAL (g)**	**1055.05**	**858.15**	**869.89**
**kcal**			
Protein	716	716	716
Carbohydrate	2840	1422.4	1422.4
Fat	405	1822.5	1822.5
** TOTAL Kcal**	**3961**	**3960.9**	**3961**
** Kcal/g**	**3.75**	**4.62**	**4.62**

Adapted from Shahinozzaman et al. [16].

### Animals and sample size

All animal procedures were in accordance to a protocol approved by the Institutional Animal Care and Use Committee (IACUC) of the University of Maryland. Thirty-six (36) male C57BL/6J mice (Jackson Labs, Bar Harbor, Maine, USA) at 8 weeks age were singly housed in controlled environmental conditions (22°C), 12-hour light dark cycle in shoebox cages containing corncob bedding. Mice had ad *libitum* access to food and water. A sample size of n = 9 for each diet group was used based on a power analysis in our previous animal study on obesity induced insulin resistance which showed 8 to be a number sufficient to generate statistically significant results.

### Randomization and feeding regime

After one-week baseline feeding of all mice on the LFD, mice were randomized based on body weight and the homeostatic model assessment of insulin resistance (HOMA-IR) into four experimental groups and fed the following diets for 12 weeks: (1) LFD, (2) HFD, (3) HFD containing kale vegetable (HFKV) and group (4) was fed the HFD diet for 6 weeks and then switched to the HFKV diet for the next 6 weeks (HF-HFKV). Food intake (weight of food administered minus the leftover and spillage) and body weight were monitored and recorded twice every week.

### Determination of insulin sensitivity

Fasting (6h) plasma glucose and insulin were determined at baseline and after 6 and 12 weeks of feeding from blood collected by mandibular bleeding. Insulin levels determined by a mouse ELISA kit (Crystal Chem, Downers Grove, IL) and blood glucose determined by a portable glucometer (Milipitas, CA, USA) were used to calculate the insulin resistance index (HOMA-IR) using the formula:

HOMA‐IR=fastingglucose(mg/dL)xfastinginsulin(ng/ml)/405.

### Tissue collection and processing

Following 12 weeks feeding, animals were anesthetized by isoflurane inhalation, terminal blood collected by heart puncture followed by euthanasia by cervical dislocation. Serum was separated and stored at -80°C. The weight of each abdominal fat pad (epididymal, perirenal, and retroperitoneal) was determined and summed as total abdominal fat. All issues collected were snap frozen in liquid nitrogen and later stored at -80°C pending further analysis.

### Histopathology

Tissues were either fixed in 10% buffered formalin or snap frozen in liquid nitrogen before preparation for histopathology to determine lipid accumulation or inflammation markers. Adipose tissue (epididymal) formalin fixed samples were dehydrated in alcohol and embedded in paraffin before sectioning to 2–3 μm thickness and subjecting them to Hematoxylin and Eosin (H&E) staining. Snap frozen liver samples were sectioned to 3–4 μm thickness and stained with oil red O (ORO) without prior fixation. Stained sections were examined and photographed under a light microscope (Zeiss Axioscope) at 40X magnification. At least 8 adipose tissue sections from three different animals in each group were analyzed and graded for cell size and inflammation markers. At least 8 liver sections from three different animals in each group were analyzed and graded for fat deposition by counting fat droplets.

### Analysis of triglycerides in liver and colon fecal samples

Triglycerides were analyzed in liver to determine lipid accumulation and also in colon fecal contents to determine amount of fat that escapes digestion or absorption in the small intestines. A triglyceride kit (Cayman Chemical, Ann Arbor MI) was used. ND40 reagent containing protease inhibitors was used to homogenize colon fecal samples (100 mg) or liver (150 mg). After centrifuging (10,000 rpm) at 4°C, the supernatant was diluted 3-fold and reacted with lipoprotein lipase to release glycerol and fatty acids. The glycerol was quantified by a colorimetric enzymatic reaction according to the kit manufacturer’s instructions.

### Serum analysis

To determine extend of dyslipidemia, total triglycerides in serum were quantified using a colorimetric kit (Cayman Chemical, Ann Arbor, MI) according to manufactures specifications. Serum LDL cholesterol and HDL cholesterol were quantified using mouse ELISA kits. Endotoxin (LPS) levels) and the chemokine MCP-1 (Ccl2) were quantified using a mouse ELISA kits. All ELISA kits were from (Cusabio, Wuhan China).

### RNA extraction and cDNA synthesis

To extract RNA for gene expression studies, about 50 mg of gastrocnemius skeletal muscle and 100 mg of the epididymal fat pad were disrupted in TRIzol with bead beating using the FastPrep-24 (MP Biomedical, Solon, Ohio) before extracting and purifying RNA using the RNAeasy mini kit (Qiagen, German Town, MD). The RNA quantity and quality were determined using the Qubit 4 fluorometer (Thermofisher Scientific, Rockville, MD) before cDNA synthesis using the RT^2^ first strand kit (Qiagen, German Town, MD) with 1000ng as starting RNA per sample.

### Analysis of inflammation in adipose tissue by RT^2^ profiler PCR array

To identify inflammation markers in adipose tissue (epididymal fat pad), the differential expression of 84 genes that encode inflammatory chemokines or cytokines was determined using the configured RT2 Profiler TM PCR Array (Qiagen, German Town, MD). About 1000 ng of cDNA from adipose tissue was mixed with SYBR green qPCR master mix and the mixture was then aliquoted in wells of the RT^2^ profiler PCR array. The array was centrifuged for 1 minute at 3000 rpm at room temperature before qPCR. Cycling conditions on the CFX 96 (Bio-rad, Hercules CA), were one cycle of 95°C for 1 minute, 40 cycles of 15 seconds at 95°C and 60°C for 60s. The threshold cycle (Cq) values for each gene and that of glyceraldehyde-3-phosphate dehydrogenase (GAPDH) as reference gene were used to calculate ΔΔCq values and relative gene expression was determined by calculating 2^-ΔΔ*CT*^. The expression of β-actin was used as the endogenous control. For genes that were downregulated, a reciprocal of 2^-ΔΔCT^ was calculated. Results were also analyzed by the GeneGlobe data analysis center (Qiagen, German Town, MD).

### Analysis of target genes in skeletal muscle, adipose tissue by qPCR

Primer sets (IDT Technologies, Coralville IA) were used for analyses of gene expressionWe selected genes that are involved in adipogenesis, lipogenesis and fatty acid oxidation. All these processes may impact diet induced obesity. RT-PCR cycling conditions on the CFX 96 (Bio-rad, Hercules CA), were 2 min at 50°C and 2 min at 95°C, followed by 40 cycles of two-step PCR denaturation at 95°C for 15 secs and annealing extension at 60°C for 1 min. Duplicate assay samples contained 10 ng cDNA and 6 μmol/L primers in 2× PowerUp SYBR Green Master Mix (Thermofisher Scientific in a final volume of 20μL. Means of duplicates were taken, and relative amount of target mRNA was normalized to β-actin levels as an endogenous control gene. Data were analyzed according to the 2^−ΔΔCT^ method and fold difference among groups was calculated.

### Analysis of proteins by western blotting and Elisa kits

Protein expression of key genes that were significantly different after qPCR were analyzed by western blotting. About 50 mg gastrocnemius muscle and 100 mg epidydimal fat were separately dissected and homogenized in buffer (1% Triton X-100, 20 mmol Tris (pH 75), 2.5 mmol sodium pyrophosphate, 150 mM NaCl, 1 mmol EGTA, 1 mmol sodium vanadate, 2 mM beta-glycerophosphate, 1 μg/ml leupeptin, 1 ul/ml aprotinin, 1 ul/ml PMSF) using FastPrep-24 (MP Biomedical, Solon, Ohio). Samples were centrifuged (12000 rpm, 10 min at 4°C) and protein concentrations of the supernatant determined by the Pierce BCA Protein Assay Kit (ThermoFisher Scientific, Carlsbad, CA, USA). Supernatants (40 μg) were resolved by SDS-PAGE and subjected to standard immunoblotting. Protein abundance was detected with mouse specific antibodies against CD11c, F4/80, PPARα, PPARγ, FIAF, Adiponectin (Cell signaling, Danvers MA, USA) and normalized by β-Actin. Optical densities of protein bands were analyzed using Image J. Data are expressed as the fold difference of the LFD control.

### Statistical analyses

Data are expressed or graphed as mean ± SEM. Statistical differences between two groups were determined using the unpaired two tailed student T-Tests. Data sets with more than two groups were analyzed by a one way ANOVA using SAS software version 9.4. (SAS Institute, Cary, North Carolina; 2013) for analysis of variance between treatments. Main effects were considered significant at *P* ≤ 0.05. Data was analyzed for outliers using studentized residuals. Significant differences observed were followed up using the Tukey’s test of multiple comparisons.

## Results

### Nutrition analysis of kale oven dried powdered sample

A comprehensive proximate analysis showed that the oven dried powdered leaves of kale sample used for this study contained 4.65% moisture, 8.71% proteins, 73.9% carbohydrates, 1.88% total fat, and 10.87% Ash. Among the carbohydrates 37.8% was insoluble fiber while 6.2% was soluble fiber. Details are shown in S1 Table. Among the total fatty acids in the lipid fraction, trans-Linolenic, linoleic, linolenic, palmitic acid and oleic acid constituted 24.7%, 12.9%, 34.3%, 13.0% and 10.1% respectively. Details are shown in S2 Table.

### Food consumption, energy intake, and body weight

**Fig 1:**
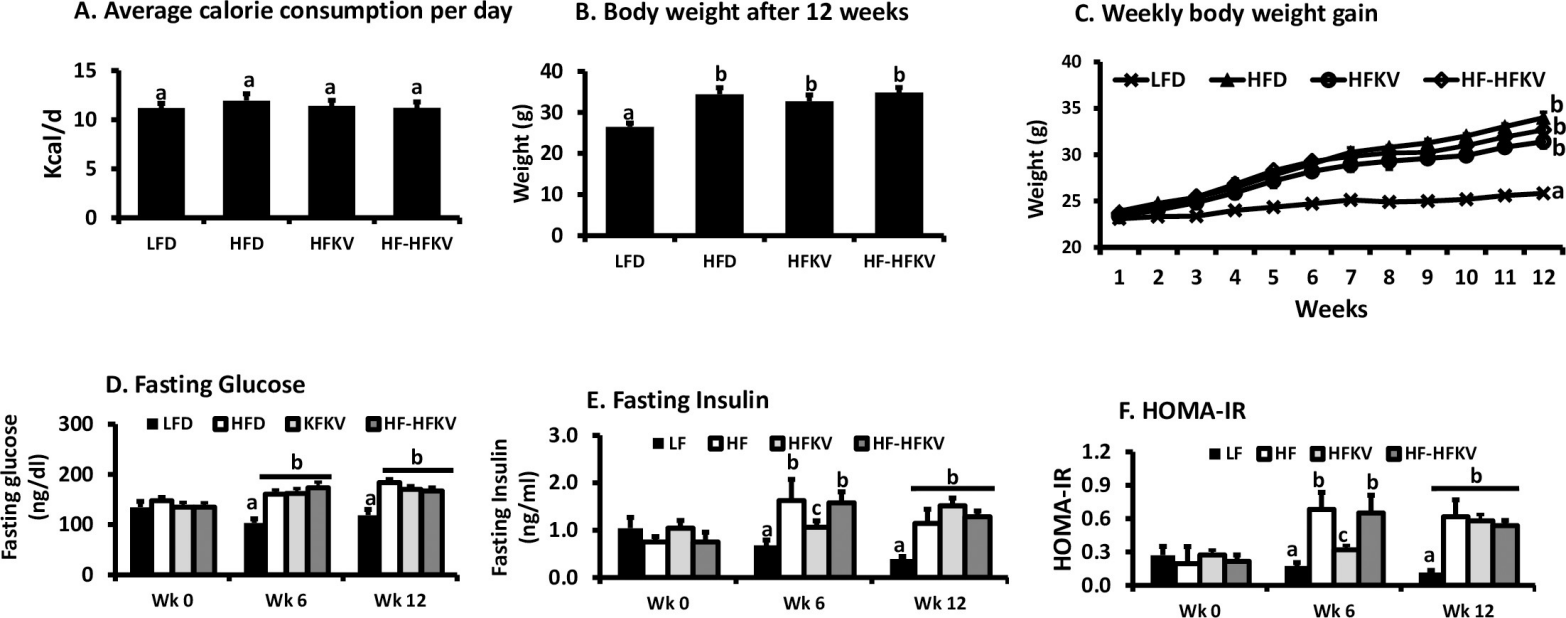
Supplementation with kale did not attenuate HFD diet induced weight gain and insulin resistance. Mice were fed isocaloric diets for 12 weeks and data was subjected to an ANOVA. **(A).** The calculated calorie consumption per day was not different among all groups (P = ns; n = 9). **(B).** The HFD induced greater body weight gain compared to LFD. Supplementing the HFD with kale from beginning of the study did not lower weight gain (P = ns; n = 9). Supplementing the HFD diet with kale after 6 weeks on HFD did not reduce weight gain compared to HFD group (P = ns; n = 9). **(C).** Weekly body weight gain was increased in the HFD group. Weight gain in both groups fed with kale was not different from that of the HFD group (P = ns; n = 9). Fasting glucose and fasting insulin were determined by glucometer and ELISA test respectively. **(D).** At week 0 (baseline), all groups had similar fasting glucose. By week 6 the HFD diet induced higher fasting glucose and kale had no effect. By week 12, the HFD fed group and HFKV groups had the same levels of fasting glucose (P>0.05; n = 8–9). **(E).** At baseline, all groups had similar fasting Insulin. By week 6 the HFD diet induced higher fasting glucose in the two groups and group 3 fed with kale had significantly lower insulin levels compared to the HF group. By week 12, the HFD fed group and the two groups fed kale had similar levels of fasting insulin (P>0.05; n = 8–9). **(F).** At baseline, all groups had similar HOMA-IR values. By week 6, the HFD diet induced higher HOMA-IR and group 3 fed with kale had significantly lower HOMA-IR (P<0.05; n = 8–9). By week 12, the HFD fed group and groups fed HFD supplemented with kale had similar levels of HOMA-IR (P>0.05; n = 8–9). ‘a’ Different letters indicate significant differences after ANOVA at P<0.05.

Weekly food consumption and the calculated energy intake per day were not different between the four groups (P>0.05; n = 9) (Fig 1A). As expected, the HFD fed mice gained significantly higher weight compared to the LFD group (P<0.05). The HFKV diet lowered body weight numerically but, the value was not statistically different from that of the HFD diet (P>0.05) (Fig 1B and 1C). Switching the diet from HFD to HFKV after 6 weeks (group 4) also had no significant effect on body weight (P>0.05) (Fig 1B and 1C).

### Insulin resistance

As expected, mice fed the HFD diet had higher fasting glucose compared to those fed the LFD. At weeks 6 and 12, fasting glucose in the two HFKV groups was similar to that of the HFD group (P>0.05) (Fig 1D). At week 6, mice fed the HFD had significantly higher fasting insulin compared to those fed the LFD (P<0.05) while fasting insulin in group 3 fed HFKV was lower than that of the HFD group (P<0.05). At week 12, fasting insulin in the two HFKV groups was not different from that of the HFD group (P>0.05) (Fig 1E). Feeding the mice HFKV over 12 weeks or introducing HFKV only in the last 6 weeks after inducing obesity with HFD did not lower fasting insulin compared to that of mice fed HFD only (Fig 1E).

At week 6, both HFD groups had significantly higher HOMA-IR compared to the LFD fed (P<0.05). The HFKV fed group had a lower HOMA-IR compared to the HFD group (P<0.05). At week 12, the groups fed HFKV diet both had the same HOMA-IR compared to the HFD group (P>0.05) (Fig 1F). Feeding HFKV over 12 weeks or introducing HFKV in the last 6 weeks after inducing obesity with the HFD had the same effect on HOMA-IR by the end of the study (Fig 1F).

#### Adipose tissue fat accumulation and histological features

**Fig 2:**
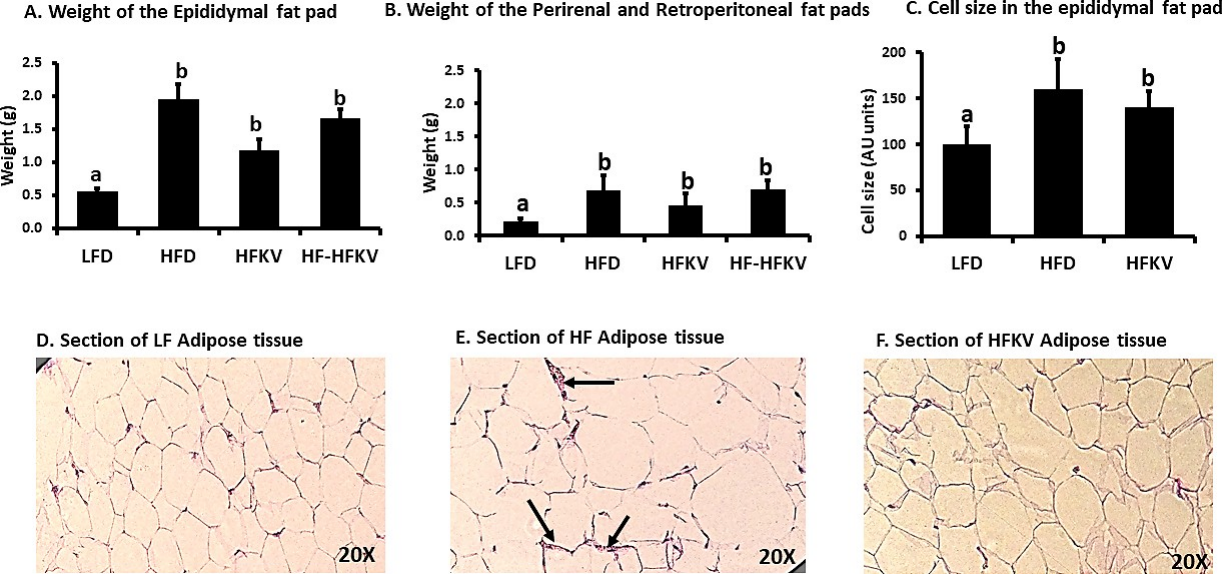
Supplementation with kale vegetable did not lower fat accumulation but reduced inflammatory markers in adipose tissue. **(A and B).** The weight of the epididymal fat pad and combined weight of perirenal and retroperitoneal fat pads were higher in HFD compared to LFD fed group (P<0.05; n = 9). Supplementing the HFD diet with kale from beginning of the study or after 6 weeks of HFD feeding did not lower fat pad weights compared to the HFD diet (P>0.05; n = 9) after ANOVA. **(C).** Cell size of epididymal fat on H and E stained sections was determined by measuring cross-sectional width of the cells at 40X magnification and standardizing LFD group size to 100. HFD diet increased cell size. The cell size of HFD and HFKV was not different (P>0.05; n = 9) after ANOVA. **(D-F).** H and E stained sections of the epididymal fat pad showed that LFD group had smaller adipocytes in size with no markers of inflammation visible. The HFD sections showed larger adipocytes and presence of crown like structures the markers of inflammation (shown by arrow). The HFKV sections showed large adipocytes but no crown like structures. ‘a’ Different letters indicate significant differences after ANOVA at P<0.05.

Summing up the weight of the fat pads (epidydimal, retroperitoneal and perirenal) for each mouse revealed that the HFD induced fat accumulation significantly higher than that in the LFD group (P<0.05; n = 9). Fat pad weights of the HFKV fed group was numerically lower than that of the HFD group but the difference was not statistically different (P>0.05). Switching the diet from HFD to HFKV in group 4 also did not reduce fat pad weight significantly (P>0.05) (Fig 2A and 2B). Epididymal tissue from mice fed the HFD had larger adipocytes (Fig 2C) and more crown like structures (histologic hallmarks of the proinflammatory process) were visible (Fig 2D and 2E). We based the observation after undergoing training in histology by a trained pathologist. Epididymal tissue of HFKV also had bigger adipocytes compared to LFD fed mice but, crown like structures were not observed (Fig 2F).

### Liver weight and lipids

**Fig 3:**
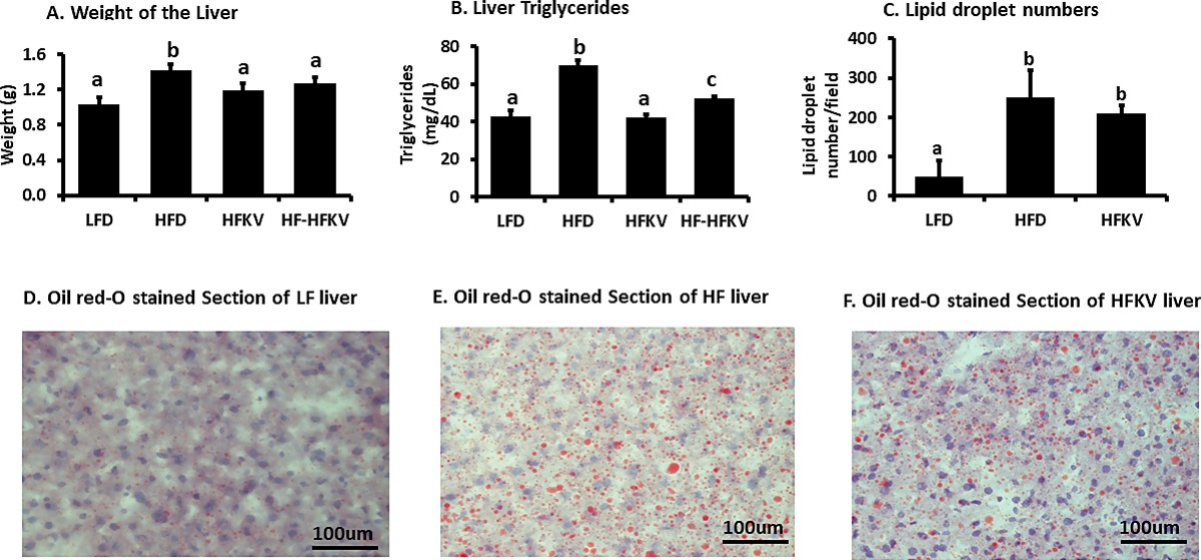
Supplementation with kale lowered liver weight and liver triglycerides but not liver lipid droplet number. **(A).** The weight of the liver was higher in HFD compared to LFD fed group (P<0.05; n = 9). Supplementation of the HFD diet with kale from beginning of the study or after 6 weeks of HFD feeding lowered liver weight to levels not different from those of the LFD (P<0.05; n = 8–9 after ANOVA). **(B).** Amount of liver triglycerides was higher in HFD compared to LFD fed group (P<0.05; n = 9). Supplementation of the HFD diet with kale from beginning of the study or after 6 weeks of HFD feeding lowered liver triglycerides to levels similar to those of the LFD (P<0.05; n = 8–9 after ANOVA). **(C).** Quantification of fat droplet number in liver sections at 40X magnification (P<0.05; n = 8–9). **(D-F).** ORO stained liver slides from HFD fed mice showed highly increased lipid droplets compared to the LFD group. The lipid droplet number in HFKV group was not different from that of the HFD group. ‘a’ Different letters indicate significant differences after ANOVA at P<0.05.

**Fig 4:**
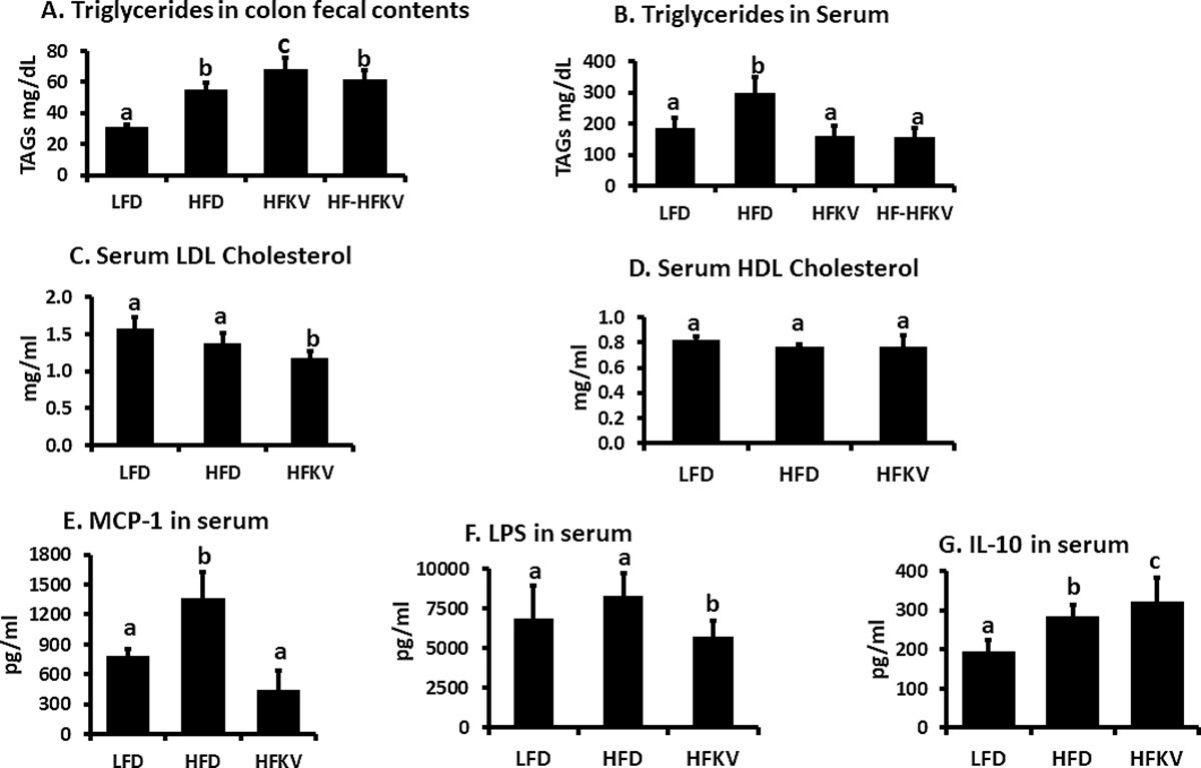
Supplementation with kale lowered serum TAGs, LDL cholesterol, endotoxemia and inflammation markers. TAGs were analyzed by a colorimetric kit. LDL and HDL cholesterol were quantified by Elisa kits. All data was subjected to after ANOVA. **(A).** TAGS in colon fecal samples were higher in HFD compared to LFD fed group (P<0.05; n = 9). TAG levels in HFKV samples were higher than those in HFD and LFD (P<0.05; n = 9). **(B).** TAG levels in serum were higher in HFD compared to LFD fed group (P<0.05; n = 9). TAG levels in HFKV samples were lower than those in HFD (P<0.05; n = 9). **(C and D).** LDL cholesterol levels in HFKV samples were lower than those in HFD and LFD (P<0.05; n = 9). HDL cholesterol levels were not different between the 3 diet groups (P = ns; n = 9). LPS, MCP-1 and IL-10 levels in serum were quantified using mouse ELISA kits. All data was subjected to ANOVA. **(E)** The HFD increased levels of LPS compared to LFD (P<0.05; n = 9). The HFKV diet attenuated the increase in LPS to levels below those of both the HFD diet and LFD (P<0.05; n = 9). **(F)** The HFD diet increased levels of MCP-1(Ccl2) compared to LFD (P<0.05). The HFKV diet attenuated the increase in MCP-1 to levels below those of both the HFD diet and LFD diet groups (P<0.05; n = 9). **(G)** The HFD diet increased the levels of IL-10 compared to LFD diet (P<0.05). The HFKV diet further increased IL-10 to levels higher than those of the HFD diet group (P<0.05; n = 9). ‘a’ Different letters indicate significant differences after ANOVA and Tukeys test of multiple comparisons at P<0.05.

Both liver weight and liver triglycerides were significantly higher in the HFD fed mice compared to those fed LFD (P<0.05; n = 9). Feeding HFKV for 12 weeks (group 3) or introducing HFKV in the last 6 weeks (group 4) after inducing obesity with the HFD, reduced liver weight and liver triglycerides to levels significantly lower than those of the HFD group but not different from the LFD group (P<0.05; n = 9) (Fig 3A and 3B). The ORO stained liver sections showed more lipid droplets in liver of HFD group compared to LFD (Fig 4C–4E). The amount of lipid droplets in livers of mice fed HFKV was similar to those of HFD fed mice (Fig 3E and 3F).

### Lipid analysis in serum and colon fecal samples

The HFD induced an increase in triglycerides in serum and colon fecal samples compared to the LFD (P<0.05 n = 9). The HFKV diet reduced circulating triglycerides in serum in both group 3 and 4 to levels below those of both the LFD and HFD groups (P<0.05). The HFD diet induced an increase in triglycerides in colon fecal samples compared to the LFD (P<0.05; n = 9). The HFKV diet increased triglycerides in colon fecal contents in both group 3 and 4 to levels higher than those of both the LFD and HFD fed groups (P<0.05; n = 9) (Fig 4B). Serum LDL cholesterol levels in LFD and HFD group were not different. Supplementation with kale vegetable lowered LDL cholesterol level compared to both LFD and HFD groups (P>0.05). HDL cholesterol levels in all diet groups were not different (P = ns) (Fig 4).

### Endotoxemia and inflammation markers in serum

The HFD increased ccl2 (MCP-1) concentrations in serum compared to LFD. MCP-1 concentration was significantly lower in HFKV diet group compared to HFD and also compared to that of LFD group (P<0.05; n = 9) (Fig 4A). The HFD diet increased LPS concentrations in serum. LPS concentration was significantly lower in HFKV diet group compared to HFD and significantly lower in HFKV diet group compared to LFD (P<0.05; n = 9) (Fig 4B). The HFD diet increased IL-10 concentrations in serum. The HFKV diet further increased serum IL-10 to levels higher than those of both the HFD and LFD groups (Fig 4C).

### Inflammation markers in adipose tissue by qPCR

**Fig 5:**
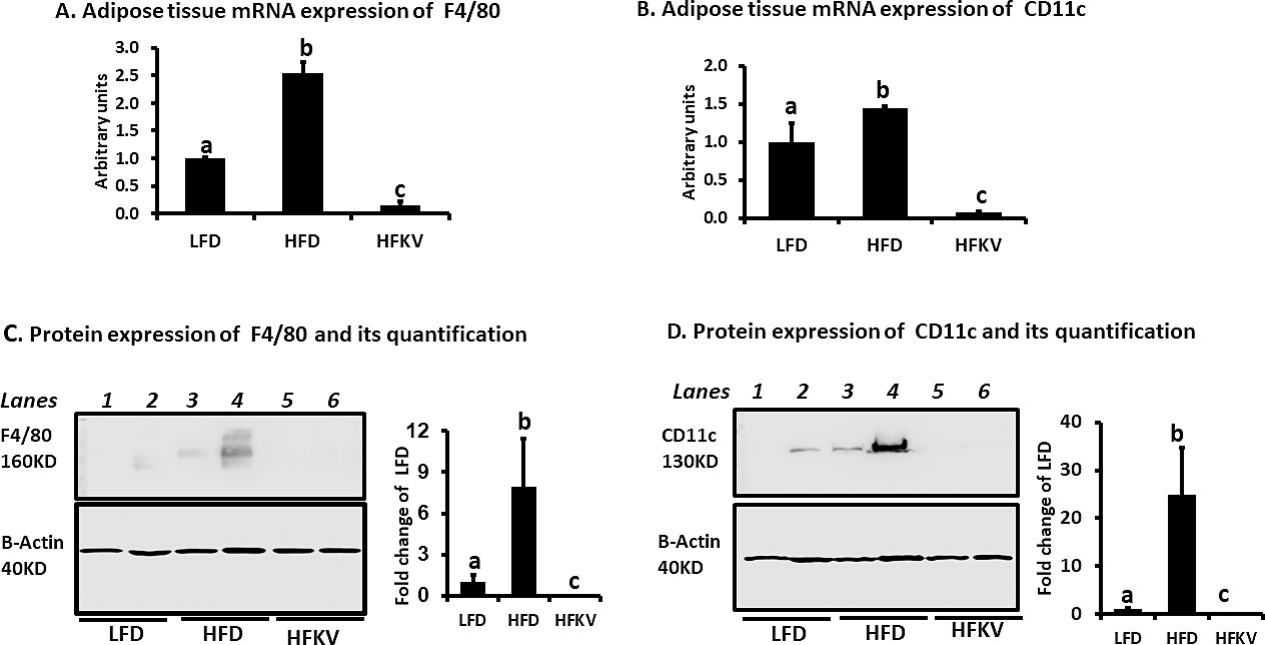
Supplementation with kale reduced adipose tissue markers of inflammation. Quantitative PCR and western blotting showed that: **(A).** The HFD increased gene expression of F4/80 compared to LFD (P<0.05). The HFKV diet decreased expression of F4/80 to levels below those of both the HFD diet and LFD groups (P<0.05). **(B).** The HFD diet increased gene expression of CD11c compared to LFD diet (P<0.05). The HFKV diet attenuated the increase in CD11c to levels below those of both the HFD and LFD groups (P<0.05). **(C).** The HFD diet increased the protein expression of F4/80 compared to LFD. The HFKV diet decreased F4/80 to levels not detectable by western blotting (P<0.05). Bar chart represents quantification of the gel blot in Image-J. **(D).** The HFD diet increased the expression of CD11c compared to LFD. The HFKV diet decreased expression of CD11c to levels not detectable by western blotting (P<0.05). The bar chart represents quantification of the gel blot in Image-J. The test proteins and β-actin were determined on the same membranes but at different exposure times. Full membranes are shown in S1 Fig. ‘a’ Different letters indicate significant differences after ANOVA at P<0.05.

The HFD diet increased the gene expression of CD11c and F4/80 compared to LFD and HFKV lowered these two genes to levels below those of both the LFD and HFD groups (Fig 5A and 5B). The protein expression of both CD11c and F4/80 increased in HFD group compared to the LFD (lanes 3 and 4 vs lanes 1 and 2). The HFKV diet prevented HFD increase in CD11c and F4/80 (lanes 5 and 6 vs lanes 3 and 4 in Fig 5C and 5D). These proteins were below levels of detection by western blotting in the group fed HFKV.

### Gene expression of adipose tissue inflammatory cytokines and receptors by RT2 Profiler TM PCR Array

**Fig 6:**
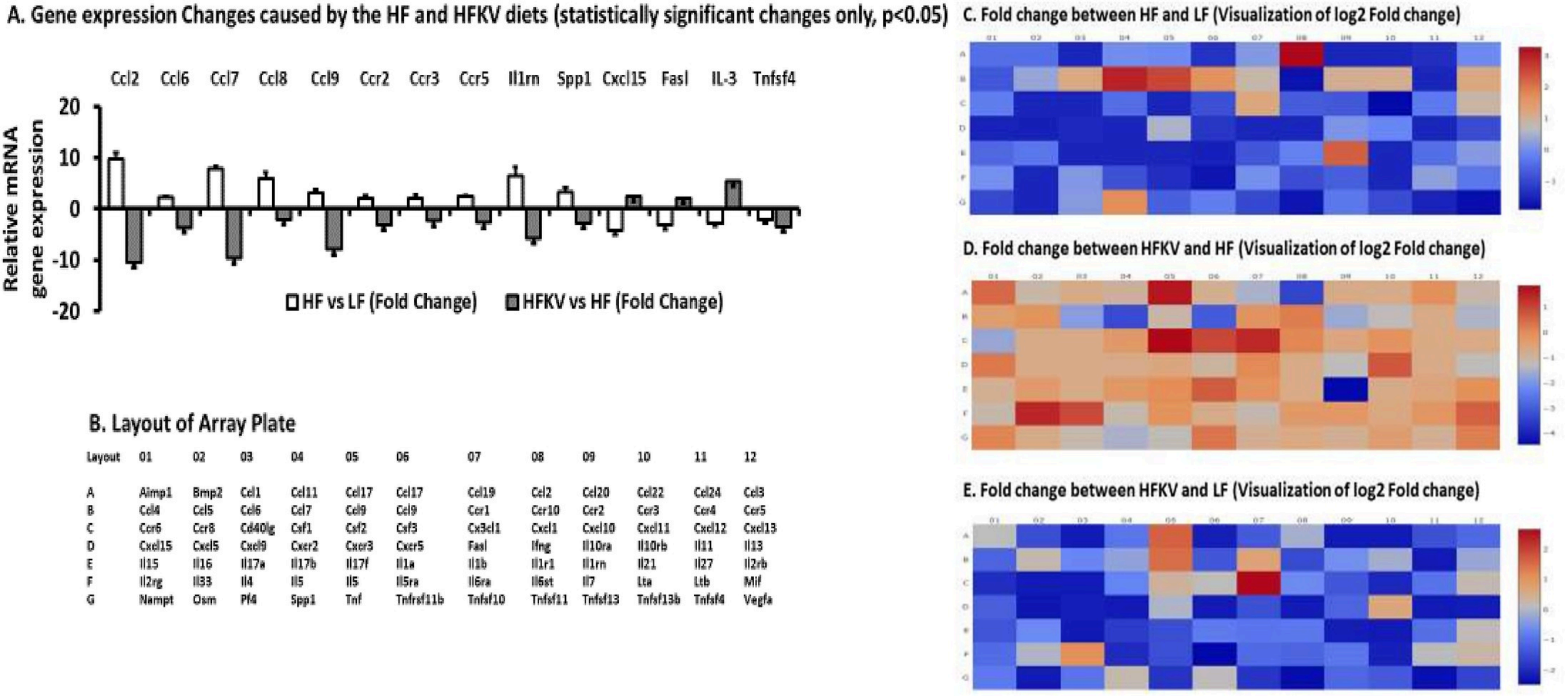
Fold change in gene expression of inflammatory cytokines, chemokines and receptors in adipose tissue. cDNA was synthesized using the RT^2^ first strand kit and using the RT^2^ Profiler TM PCR Array, the differential expression of the chemokines, cytokines and receptors was determined. **(A).** The HFD diet increased ten chemokines and chemokine receptors by over 3.0-fold and the HFKV diet reduced these. The HFD reduced 3 cytokines and the HFKV diet increased them. All genes shown were changed by over 3.0-fold and were statistically different at P<0.05 after paired T tests. **(B).** The layout of the PCR array plate. **(C-E).** Heat maps displaying changes between the HFD and LFD, HFD and HFKV and HFKV and LFD respectively.

Fig 6 shows the relative mRNA expression of the LFD group (as control) relative to the HFD group, HFD (as control) relative to HFKV, and the LFD (as control) relative to the HFKV group. Fig 6A and 6B shows values of genes that changed by more than 3.0-fold and were significantly different at P<0.05. The other observations for all 84 genes are shown in the heat maps (Fig 6C–6E) and S3 Table which has the raw data for the heat maps.

### Gene and protein expression in adipose tissue skeletal muscle

**Fig 7:**
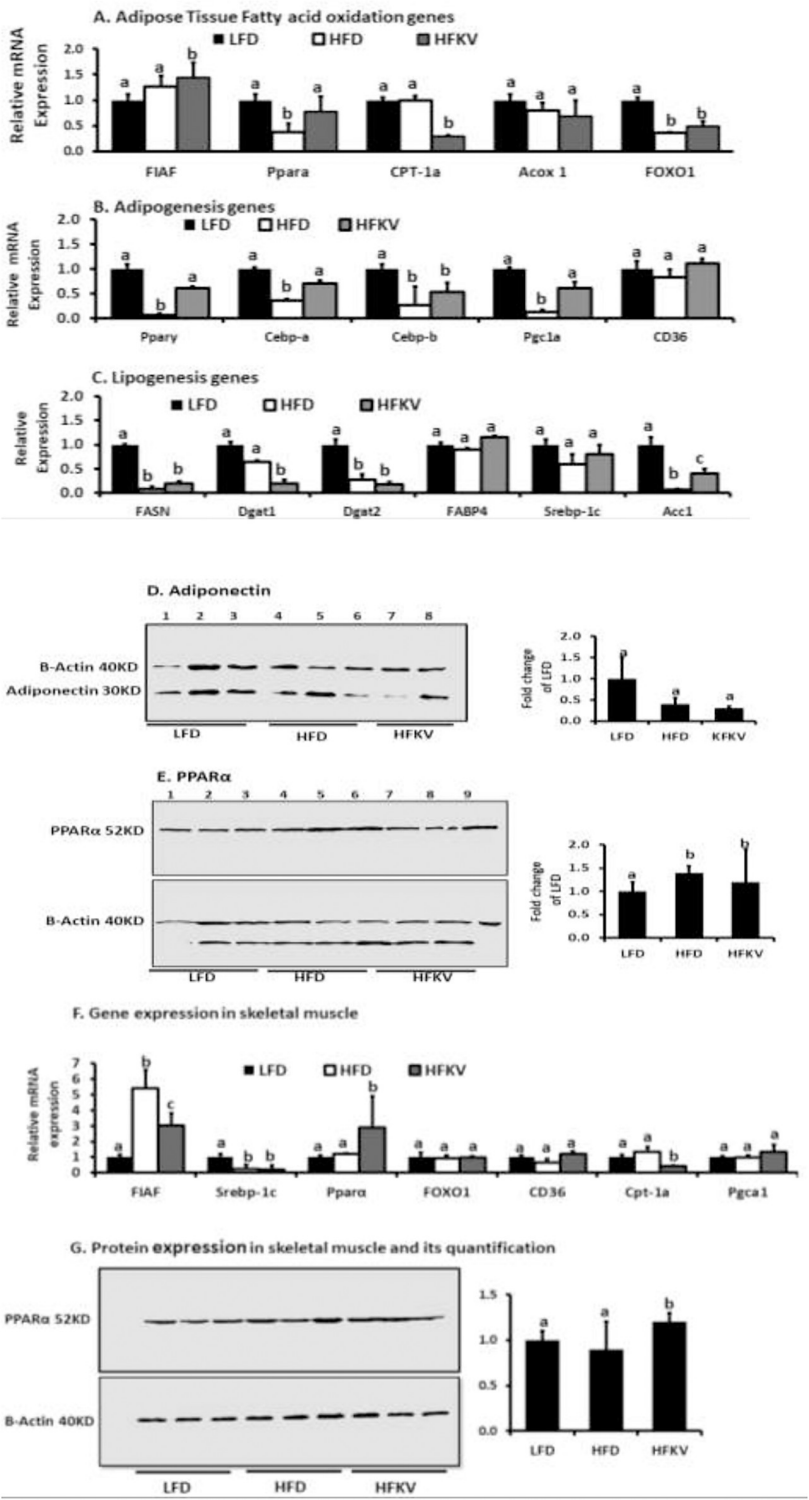
Gene expression and protein expression in adipose tissue and skeletal muscle. Gene expression was determined by qPCR and Cq values for each gene and that of β-actin as reference gene were used to calculate gene expression by calculating 2^-ΔΔ*CT*^. The bar charts next to the western blots shows quantification of the western blot band in image J and after ANOVA. **(A).** Genes involved in fatty acid oxidation **(B).** Genes involved in adipogenesis. **(C).** Markers of adipose tissue lipogenesis. **(D).** Protein expression of Adiponectin. HFD decreased adiponectin expression and HFKV had no effect (P = ns). (**E).** Protein expression of PPARα. HFD decreased PPARα expression and HFKV had no effect. **(F).** Genes involved in fatty acid oxidation in skeletal muscle. HFKV lowered the expression of FIAF and increased that of PPARα (P<0.05). **(G).** Protein expression in skeletal muscle. The bar chars represent quantification of the protein standardized by expression of β-Actin. Expression of PPARα was higher in HFKV diet compared to the LFD and HFD (P<0.05). The test proteins and β-actin were determined on different membranes. Full blots are shown in the S2 Fig. ‘a’ Different letters indicate significant differences at P<0.05.

In adipose tissue, the HFKV diet enhanced the peroxisome proliferator-activated receptor alpha (PPARα) and reduced carnitine acyltransferase I (Cpt-1a) compared to the HFD diet; these are both markers of fatty acid oxidation. The HFKV diet had no effect on expression of fasting induced adipocyte factor (FIAF), peroxisomal acyl-coenzyme A oxidase 1 (Acox1), and Forkhead box protein O1 (FOXO1) also markers of fatty acid oxidation (Fig 7A). The HFD diet lowered the expression of genes involved in adipogenesis; peroxisome proliferator-activated receptor gamma (PPARγ), CCAAT/enhancer binding protein alpha (C/EBPa), C/EBPb, peroxisome proliferator-activated receptor gamma coactivator 1-alpha (Pgc1a) (P<0.05). The HFKV diet prevented this reduction and levels of these genes were not different from those of the LFD group. The expression of CD36 was not different between the LFD, HFD and HFKV groups (Fig 7B). Markers of lipogenesis and triglyceride synthesis like fatty acid synthase (Fasn), diacylglycerol O-acyltransferase 1 (Dgat1), Dgat2, Acetyl-CoA carboxylase (ACC1), and fatty acid binding protein 4 (FABP4) were lowered by the HF diet. The HFKV diet had no effects on most lipogenesis genes evaluated (P>0.05) but, it increased ACC1 and lowered Dgat1 (P<0.05; n = 9) (Fig 7C). A follow-up on protein expression showed that the HFD lowered adiponectin expression but HFKV diet had no significant effects (Fig 7D). No effect of diet was observed on PPARα protein expression (Fig 7E) (P>0.05). The HFKV diet significantly increased PPARγ compared to HFD and LFD (P<0.05) but had no effect on FIAF expression.

In Fig 7F we show the mRNA expression of genes that impact fatty acid oxidation in skeletal muscle. Compared to the HFD, HFKV lowered the gene expression of FIAF and Cpt-1a compared to HFD and increased PPARα (P<0.05; n = 9) but, had no effect on Pgc1a, Srebpc-1c, FOXO1, CD36, (P>0.05) (Fig 7F). Protein expression of PPARα was higher in HFKV compared to HFD (lanes 4–6 vs lanes 7–9 in Fig 7G) but no difference was observed for FIAF expression among all treatments.

## Discussion

Although kale places high on the list of ‘most healthy foods’ it remains an understudied vegetable. We show that supplementing a HFD with kale does not significantly reduce weight gain, fat accumulation and insulin resistance but, it prevents HFD diet induced endotoxemia, improves lipid metabolism by lowering serum triglycerides and LDL cholesterol and attenuates both systemic and adipose tissue specific inflammation thus contributing to metabolic health. Previous work on cruciferous vegetables focuses on broccoli and spinach. We focused on kale because it is a relatively newly popular food in the United States although it has been popular in other international cultures/diets such as the Mediterranean diet. Our negative findings on obesity phenotype are not in conflict with any previous studies in the literature. These findings had just not been shown before. Although there are claims on the benefits of kale on insulin resistance, type 2 diabetes and obesity, there is no solid data to merit such claims. The ability of kale to attenuate inflammation and endotoxemia are important key finding as this translates into better metabolic health despite lack of effects on body weight.

The bioactive phytochemicals in cruciferous vegetables including kale have been widely characterized. The health benefits are associated with glucosinolates, polyphenols, carotenoid groups, terpenoids and various indole derivatives [6–8]. Kale is also rich in vitamins, folate, potassium and fiber [9, 11]. In this preliminary study we did not characterize the phytochemical composition of kale but focused on assessing the impact of the whole vegetable as a functional food. One key initial finding was the ability of kale to improve lipid metabolism (Fig 4). In agreement with Kahlon et al. [14], we show that the HFD supplemented with kale lowered LDL cholesterol levels. The mechanism by which food components lower cholesterol is thought to be related to binding of bile acids [14]. Kale contains bile acid sequestrants, which prevent bile acid recirculation resulting in reduced fat absorption, excretion of cancer-causing toxic metabolites and cholesterol utilization to synthesize more bile acids [14]. The higher amounts of triglycerides in colon fecal samples of mice supplemented with kale is evidence that it reduces the amount of fat absorbed in the intestines (Fig 4A).

As a follow up to a previous study [16] in which we showed that kale attenuated adipose tissue specific inflammation, we performed further tests on inflammation markers both in serum and adipose. Both circulating levels of Ccl2 and those in adipose tissue were increased by the HFD but including kale in the HFD lowered these to levels below those of the HFD (Figs 4 and 6). The effect on Ccl2 was more pronounced in adipose tissue where the HFD increased its gene expression by over 9-fold and kale lowered them by over 10-fold (Fig 6). Ccl2 is a macrophage and monocyte recruiter in adipose and its downregulation likely contributed to the observed lack of crown like structures (hallmark features of inflammation) in adipose tissue of mice fed with kale (Fig 2). By reducing expression of Ccl2, kale components may exert anti-inflammatory effects through modulation of toll-like receptor 4 signaling and modification of cytokine macrophage migration. The abundance of the mRNA of Ccl2 has been shown to positively correlate with that of CD11c in visceral adipose of obese humans with metabolic syndrome compared with lean humans [18]. In agreement with this we show that the HFD induced a more than 2-fold increase in CD11c compared to the LFD group. Including kale in the diet prevented this and down regulated CD11c and F4/80 another marker of inflammation to levels lower than those of both HFD and LFD diet groups (Fig 5). Macrophages that expresses CD11c produce high amounts of pro-inflammatory cytokines, are recruited to adipose and muscle tissue and are linked to the development of obesity-associated insulin resistance. When CD11c^+^ cells are depleted in obese mouse models, it leads to normalization of insulin sensitivity and a marked decrease in gene expression and protein levels of inflammatory markers [18–20]. However, in this study, insulin resistance was not improved.

Further evidence of the impact of kale on inflammation is on the reduction in the gene expression of a battery of chemokines and chemokine receptors compared to the HFD (Fig 6). This positive result corroborates those of several others who have shown that the phytochemicals in cruciferous vegetables mainly impact health through anti-inflammatory mechanisms [18–23]. While, the HFD diet decreased Cxcl15, Fas ligand (TNF superfamily, member 6) and IL-3, the HFKV diet prevented this decrease and infact increased the gene expression when compared to the HFD diet. Cytokine IL-3 is a hematopoietic factor required for survival and proliferation of hematopoietic progenitor cells and improves the body’s natural response to disease as part of the immune system. When Fasl binds to its Fas receptor, it triggers apoptosis that plays a pivotal role in the maintenance of immune system homeostasis [21–24]. Thus, HFKV not only prevented the increase of inflammatory cytokines and chemokines but also prevented the decrease in genes or cytokines that are beneficial for immune homeostasis. This result also corroborates with studies that have shown that sulforaphane (a component of kale) induces the expression of several cytoprotective genes that are key in cellular defense mechanisms [25, 26].

We also studied the effect of kale in attenuating endotoxemia because it is related to both inflammation and insulin resistance. Obesity leads to the low-grade elevation of gut derived LPS (metabolic endotoxemia) [27]. Endotoxemia may lead to low-grade systemic inflammation and leads to the transition from metabolically healthy obesity to metabolic syndrome characterized by elevated inflammatory cytokines and macrophage infiltration into adipose tissue [27, 28]. When bound to its receptor Toll-Like Receptor 4, LPS stimulates whole-body and tissue specific metabolic perturbations by initiating a signaling cascade that results in inflammatory pathways and further promotes obesity and insulin resistance [27, 28]. The HFD increased amounts of LPS in serum and kale attenuated it and infact reduced LPS to concentrations below those found in both HFD and LFD control groups (Fig 6). Because LPS is produced by gut bacteria [27], it is likely that kale impacts the gut microbiota thus changing the amount of bacterial species or taxa that predominantly produce LPS or cause changes in gastrointestinal barrier function. This result corroborates previous studies that have shown that sulforaphane attenuates LPS effects [29, 30] and inhibits LPS-induced monocyte adhesion via suppression of intercellular adhesion molecule-1 (ICAM-1) and by activating transcription factor Nrf2 [26, 27].

The kale supplemented diet upregulated genes and proteins involved in adipogenesis including PPARγ. Phytochemicals in cruciferous vegetables have been shown to enhance PPARγ stability through aryl hydrocarbon receptors (AhR) and this enhances adipogenesis. AhR regulates PPARγ stability and AhR–PPARγ interaction is a potential therapeutic target for metabolic diseases [31]. The lack of positive effects on body weight and fat accumulation may be connected to the fact that we did not observe a significant effect on the expression genes or proteins involved in fatty acid transport and oxidation in both adipose tissue and skeletal muscle except for PPARα in skeletal muscle (Fig 7). In our previous study [16] we showed that kale supplementation attenuated HFD induced increases in the representation of bacteria from phylum Proteobacteria particularly class Gammaproteobacteria and family Enterobacteriaceae. Gammaproteobacteria benefit from the effects of inflammation and contain molecular components that directly enhance the inflammatory response. Kale supplementation also increased representation of Coriobacteriaceae a family identified as a potential biomarker of improved health outcomes after interventions such as exercise or bariatric surgery. Thus, the impact of lower inflammation and endotoxemia may be mediated through impacts of kale on the microbiota composition.

In conclusion, like other cruciferous vegetables, consumption of kale results in the prevention of systemic and tissue specific inflammation and reduces expression of macrophage and inflammation markers in adipose tissue but this effect is not sufficient in preventing insulin resistance and adiposity. The majority of beneficial effects were found when kale was administered from the beginning of the HFD diet regime as a preventive measure (group 3) rather than when it was admistered to reverse HFD fat effects after 6 weeks (group 4) We therefore did not include the tissues from group 4 on further molecular biology tests. Because the arbitrary dose selected in this preliminary study was rather high the next study will involve a dose response to find the lowest dose at which kale prevents inflammation and endotoxemia. The gene panel shown in Fig 6 may represent a putative biomarker set, a target set of genes that are impacted by components of kale vegetable and should be pursued for further mechanistic studies. Kale may provide effective protection from inflammation and endotoxemia without substantial disruption of the diet. The limitation of our study is that it was designed as a comparative study between the HFD and the HFD supplemented with kale to search for evidence of efficacy, but we do not prove molecular mechanism.

## Supporting information

S1 Fig Supplementation with kale reduced adipose tissue markers of inflammation. Quantitative PCR and western blotting showed that: **(A).** The HFD diet increased the protein expression of F4/80 compared to LFD diet. The HFKV diet decreased expression of F4/80 to levels not detectable by western blotting (P<0.05). The positive control was protein extract from macrophages. **(B).** The HFD diet increased the expression of CD11c compared to LFD. The HFKV diet decreased expression of CD11c to levels not detectable in HFKV samples by western blotting (P<0.05). The positive control was protein extract from macrophages. The test proteins and β-actin were determined on the same membranes at different exposure times. The black line separates molecular markers. Different letters indicate a significant difference among treatments (p<0.05).
(TIFF)

S2 Fig Gene expression and protein expression in adipose tissue and skeletal muscle. **(A)** Protein expression of Adiponectin in adipose tissue. HFD decreased adiponectin expression and HFKV had no effect (P = ns). (**B).** Protein expression of PPARα in adipose tissue. HFD decreased PPARα expression and HFKV had no effect. **(C).** β-actin protein expression. **(D).** Expression of PPARα in skeletal muscle was higher in HFKV diet compared to the LFD and HFD (P<0.05). The bar chars represent quantification of the protein standardized by expression of β-Actin. The test proteins and β-actin were determined on different membranes.
(TIFF)

S1 Table A comprehensive nutrient analysis of the oven dried powdered kale sample. (DOCX)

S2 Table Fatty acid profile of the kale powdered vegetable sample. (DOCX)

S3 Table Gene expression (fold change) between treatments of all 84 genes after analyses by GeneGlobe software as shown in the heat maps (Fig 6C–6E). *statistically significant changes at (p<0.05).
(DOCX)
