# Supplementary material for: Kale supplementation during high fat feeding improves metabolic health in a mouse model of obesity and insulin resistance
Source: PLoS One. 2021 Aug 25;16(8):e0256348. doi: 10.1371/journal.pone.0256348 (PMC8386848; doi:10.1371/journal.pone.0256348)
Supplement: S1 Table — (DOCX) [file pone.0256348.s003.docx]

**S1 Table. Nutrient Analysis of leaves and stems of kale vegetable.**

| **Component** | **Amount %** | **Analytical Method used** |
| --- | --- | --- |
| ***Proximate Analysis*** |  |  |
| Ash | 10.87 | AOAC: 923.03 |
| Carbohydrates | 73.9 | By Calculation |
| Protein (6.25) | 8.71 | AACC 46-30; AOAC 992.15 |
| Total Fat | 1.88 | AOAC: 996.06 |
| ***Fiber*** |  |  |
| Insoluble Dietary Fiber | 37.8 | AOAC: 991.43 |
| Soluble Dietary Fiber | 6.2 |  |
| Total dietary Fiber | 45.1 |  |
| ***Fat*** |  | AOAC: 996.06 |
| Saturated Fat | 0.28 |  |
| Monounsaturated Fat | 0.21 |  |
| cis-cis polyunsaturated fat | 0.85 |  |
| Trans fat | 0.46 |  |
| Moisture | 4.65 | AOAC: 945.43, 934.01 |
| ***Calories per 100g***  Calories | 347.26 | By calculation |
| Calories, 2020 | 184.74 |  |
| Calories from Fat | 17 |  |
| Calories from Saturated Fat | 3 |  |
| Calories (insoluble fiber subtracted) | 196 |  |

**From Shahinozaman et al. [13].**
