# Supplementary material for: Kale supplementation during high fat feeding improves metabolic health in a mouse model of obesity and insulin resistance
Source: PLoS One. 2021 Aug 25;16(8):e0256348. doi: 10.1371/journal.pone.0256348 (PMC8386848; doi:10.1371/journal.pone.0256348)
Supplement: S2 Table — (DOCX) [file pone.0256348.s004.docx]

**S2 Table. Fatty acid profile of the leaves and stems Kale.**

| **Component Name** | **Normalized by Weight** | **% (w/w) as Triglyceride in Product** |
| --- | --- | --- |
| C-16:0 Palmitic | 12.9.0% | 0.24 |
| C-16:1 t-Hexadecenoic | 0.56% | 0.01 |
| C-17:1 Margaroleic | 0.56% | 0.01 |
| C-18:0 Stearic | 2.81% | 0.05 |
| C-18:1 Oleic | 10.1% | 0.19 |
| C-18:2 Linoleic | 12.9% | 0.24 |
| C-18:3 Linolenic | 34.3% | 0.64 |
| C-18:3t-Linolenic | 24.71 | 0.46 |
| C-22:1 Erucic | 1.11% | 0.02 |
| **Totals:** | **100%** | **1.40** |
