# Supplementary material for: Kale supplementation during high fat feeding improves metabolic health in a mouse model of obesity and insulin resistance
Source: PLoS One. 2021 Aug 25;16(8):e0256348. doi: 10.1371/journal.pone.0256348 (PMC8386848; doi:10.1371/journal.pone.0256348)
Supplement: S3 Table — *statistically significant changes at (p<0.05). (DOCX) [file pone.0256348.s005.docx]

**S3 Table. Fold Change in expression between treatments after analyses by GeneGlobe software (Qiagen, MD) *statistically significant changes at (p<0.05).**

| **Well** | **Gene Symbol** | **HF Vs LF** | **HFKV Vs HF** | **HFKV Vs LF** |
| --- | --- | --- | --- | --- |
| A01 | Aimp1 | -1.34 | 1.43 | 1.07 |
| A02 | Bmp2 | -1.35 | -2.09 | -2.82 |
| A03 | Ccl1 | -2.79 | -1.67 | -4.64 |
| A04 | Ccl11 | 1.00 | -1.90 | -1.89 |
| **A05** | **Ccl12*** | **-1.03** | **3.18** | **3.1** |
| A06 | Ccl17 | -2.47 | -1.79 | -4.41 |
| A07 | Ccl19 | 1.13 | -2.80 | -2.49 |
| A08 | **Ccl2*** | **9.69** | **-10.53** | **-1.09** |
| A09 | Ccl20 | -2.79 | -1.67 | -4.64 |
| A10 | Ccl22 | -2.77 | -1.68 | -4.64 |
| A11 | Ccl24 | -2.57 | -1.07 | -2.73 |
| A12 | Ccl3 | -1.02 | -2.10 | -2.15 |
| B01 | Ccl4 | -1.72 | -1.30 | -2.24 |
| B02 | Ccl5 | 1.26 | -1.09 | 1.16 |
| **B03** | **Ccl6*** | **2.28** | **-3.63** | **-1.6** |
| **B04** | **Ccl7*** | **7.90** | **-9.69** | **-1.23** |
| **B05** | **Ccl8*** | **5.92** | **-2.09** | **2.83** |
| **B06** | **Ccl9*** | **3.11** | **-7.95** | **-2.56** |
| B07 | Ccr1 | 1.80 | -1.10 | 1.65 |
| B08 | Ccr10 | -3.36 | 1.16 | -2.89 |
| B09 | Ccr2 | 2.09 | -3.10 | -1.48 |
| B10 | Ccr3 | 2.14 | -2.36 | -1.1 |
| B11 | Ccr4 | -2.79 | -1.67 | -4.64 |
| B12 | Ccr5 | 2.34 | -2.73 | -1.17 |
| **C01** | **Ccr6*** | **-1.17** | **-3.26** | **-3.81** |
| C02 | Ccr8 | -2.79 | -1.67 | -4.64 |
| C03 | Cd40lg | -2.79 | -1.67 | -4.64 |
| C04 | Csf1 | -1.37 | -1.21 | -1.65 |
| **C05** | **Csf2*** | **-2.79** | **3.63** | **1.3** |
| C06 | Csf3 | -1.82 | 1.98 | 1.09 |
| **C07** | **Cx3cl1*** | **2.35** | **2.68** | **6.3** |
| C08 | Cxcl1 | -1.62 | 1.05 | -1.54 |
| C09 | Cxcl10 | -1.68 | -1.53 | -2.57 |
| C10 | Cxcl11 | -3.72 | -1.10 | -4.07 |
| C11 | Cxcl12 | -1.21 | -1.60 | -1.94 |
| C12 | Cxcl13 | 1.87 | -1.64 | 1.14 |
| D01 | Cxcl15 | -2.88 | 1.20 | -2.39 |
| D02 | Cxcl5 | -2.90 | -1.67 | -4.82 |
| D03 | Cxcl9 | -2.68 | -1.65 | -4.44 |
| D04 | Cxcr2 | -2.79 | -1.67 | -4.64 |
| D05 | Cxcr3 | 1.46 | -1.50 | -1.02 |
| D06 | Cxcr5 | -2.37 | -1.96 | -4.64 |
| D07 | Fasl | -2.79 | 1.02 | -2.72 |
| D08 | Ifng | -2.79 | -1.67 | -4.64 |
| D09 | Il10ra | 1.07 | -2.42 | -2.26 |
| D10 | Il10rb | -1.06 | 1.69 | 1.59 |
| D11 | Il11 | -2.79 | -1.67 | -4.64 |
| D12 | Il13 | -1.91 | -2.37 | -4.53 |
| E01 | Il15 | -1.45 | -1.81 | -2.63 |
| E02 | Il16 | -1.26 | -1.25 | -1.57 |
| E03 | Il17a | -2.79 | -1.67 | -4.64 |
| E04 | Il17b | -2.79 | -1.19 | -3.33 |
| E05 | Il17f | -2.79 | 1.00 | -2.78 |
| E06 | Il1a | -2.90 | 1.61 | -1.8 |
| E07 | Il1b | -1.72 | -1.10 | -1.89 |
| E08 | Il1r1 | -1.13 | -1.64 | -1.85 |
| **E09*** | **Il1rn** | **4.86** | **-21.65** | **-4.45** |
| E10 | Il21 | -2.79 | -1.67 | -4.64 |
| E11 | Il27 | -1.36 | -1.48 | -2.01 |
| E12 | Il2rb | 1.14 | -1.04 | 1.1 |
| F01 | Il2rg | 1.03 | -2.12 | -2.07 |
| **F02*** | **Il3** | **-2.79** | **2.72** | **-1.02** |
| F03 | Il33 | 1.11 | 1.93 | 2.14 |
| F04 | Il4 | -1.80 | -2.17 | -3.9 |
| F05 | Il5 | -2.55 | -1.09 | -2.77 |
| F06 | Il5ra | -3.31 | -1.67 | -5.52 |
| F07 | Il6ra | 1.02 | -2.15 | -2.11 |
| F08 | Il6st | -1.86 | -1.21 | -2.25 |
| F09 | Il7 | -1.60 | -1.18 | -1.88 |
| F10 | Lta | -2.79 | -1.55 | -4.32 |
| F11 | Ltb | 1.20 | -1.15 | 1.05 |
| F12 | Mif | -1.25 | 1.53 | 1.23 |
| G01 | Nampt | -1.98 | 1.08 | -1.83 |
| G02 | Osm | -2.79 | -1.67 | -4.64 |
| G03 | Pf4 | 1.13 | -2.17 | -1.91 |
| **G04** | **Spp1*** | **3.31** | **-2.82** | **1.17** |
| G05 | Tnf | -1.54 | -2.34 | -3.59 |
| G06 | Tnfrsf11b | -1.16 | 1.25 | 1.08 |
| G07 | Tnfsf10 | -1.98 | -1.80 | -3.56 |
| G08 | Tnfsf11 | -3.27 | -1.61 | -5.26 |
| G09 | Tnfsf13 | -1.23 | -1.80 | -2.21 |
| G10 | Tnfsf13b | -1.89 | -1.31 | -2.47 |
| G11 | Tnfsf4 | -2.84 | -1.80 | -5.12 |
| G12 | Vegfa | -3.50 | 1.14 | -3.07 |
